# Insulin Signaling as a Therapeutic Target in Glaucomatous Neurodegeneration

**DOI:** 10.3390/ijms22094672

**Published:** 2021-04-28

**Authors:** Sara Al Hussein Al Awamlh, Lauren K. Wareham, Michael L. Risner, David J. Calkins

**Affiliations:** 1 Vanderbilt Eye Institute, Vanderbilt University Medical Center, Nashville, TN 37232, USA; sarah.a.al-awamlh@vumc.org (S.A.H.A.A.); lauren.wareham@vumc.org (L.K.W.); michael.l.risner@vumc.org (M.L.R.); 2 Department of Ophthalmology & Visual Sciences, Vanderbilt University School of Medicine, Nashville, TN 37232, USA

**Keywords:** glaucoma, insulin, neurodegeneration, neuroprotection, PI3K/Akt, RGC, CNS

## Abstract

Glaucoma is a multifactorial disease that is conventionally managed with treatments to lower intraocular pressure (IOP). Despite these efforts, many patients continue to lose their vision. The degeneration of retinal ganglion cells (RGCs) and their axons in the optic tract that characterizes glaucoma is similar to neurodegeneration in other age-related disorders of the central nervous system (CNS). Identifying the different molecular signaling pathways that contribute to early neuronal dysfunction can be utilized for neuroprotective strategies that prevent degeneration. The discovery of insulin and its receptor in the CNS and retina led to exploration of the role of insulin signaling in the CNS. Historically, insulin was considered a peripherally secreted hormone that regulated glucose homeostasis, with no obvious roles in the CNS. However, a growing number of pre-clinical and clinical studies have demonstrated the potential of modulating insulin signaling in the treatment of neurodegenerative diseases. This review will highlight the role that insulin signaling plays in RGC neurodegeneration. We will focus on how this pathway can be therapeutically targeted to promote RGC axon survival and preserve vision.

## 1. Introduction

Glaucoma is the leading cause of irreversible blindness worldwide. Glaucoma is associated with increased age and thus, its prevalence is expected to escalate to 111.8 million by 2040 as life expectancy also increases [1]. The disease manifests in different forms, but the common pathophysiologic link between them is the loss of the retinal ganglion cells (RGCs) and their centrally projecting axons that comprise the optic nerve. In the clinic, loss of ganglion cell axons translates to visual field loss that usually begins peripherally and advances centrally. The progressive and irreparable neurodegeneration of RGCs and their axons that define glaucoma likens it to other chronic age-related diseases of the nervous systems such as Alzheimer’s disease (AD) and Parkinson’s disease (PD) [2].

Stress caused by sensitivity to intraocular pressure (IOP) is conveyed to RGC axons at the optic nerve head (ONH), ultimately resulting in their progressive bidirectional degeneration [3]. Sensitivity to IOP remains the only modifiable risk factor. For many patients, glaucoma ravages the optic projection despite hypotensive treatments to reduce IOP to below normotensive levels. Although hypotensive therapies are the first guard against further neurodegeneration, glaucoma’s etiology involves an interplay between multiple pathogenic arms that includes neuronal, glial, and vascular dysfunction. Thus, neuroprotective therapeutics for glaucoma will rest in identifying molecular signaling pathways between visual neurons, glia, and vasculature that promote or counter homeostasis.

Based on epidemiological and experimental studies, the insulin signaling pathway is a promising target for neuroprotection in neurodegenerative diseases, including AD, PD, and glaucoma. This premise is based on the prevalence of insulin resistance and neurodegenerative disease comorbidity. Patients with type 2 diabetes mellitus (T2DM), a disease of peripheral insulin resistance, are more likely to also have AD, PD, or glaucoma. After adjusting for age and other potential confounders, diabetes and its duration have been found to be significantly associated with an increased risk of glaucoma [4,5,6,7]. Similarly, T2DM has been identified as a risk factor for AD [8].

The premise that insulin signaling could be therapeutic is strengthened by experimental studies investigating neurodegenerative diseases in models of insulin resistance. Animals with increased insulin resistance fed a high-fat diet demonstrate greater oxidative stress and dopamine depletion in the substantia nigra and the striatum, hallmark features of PD [9]. Aberrant insulin signaling in animal models of type 1 and 2 diabetes also induces AD pathology [10]. Additionally, inducing central insulin resistance in rodents using S961, a potent blocker of insulin receptors (IR), causes elevation in IOP and loss of RGCs, two significant pathological characteristics of glaucoma [11]. This indicates that insulin resistance may have an etiological role in the progression of glaucomatous neurodegeneration and thus enhancing insulin sensitivity may serve as a potential therapeutic modality for glaucoma.

## 2. Insulin Signaling in the CNS and Retina

Insulin, a 51 amino acid peptide hormone, is secreted by pancreatic islet beta cells in response to increasing levels of plasma glucose and amino acids [12]. Insulin is part of a family of peptides including insulin-like growth factors I/II (IGF-I/II) and relaxin, whose role is to maintain physiological levels of blood glucose [12]. It was thought that the CNS was insulin-independent; however, the expression of the insulin receptor is evident in multiple regions of the brain, including the retina [13,14]. The discovery of insulin in the CNS in extracts of whole rat brain raised questions about its origin [15,16]. The increase in cerebrospinal fluid (CSF) insulin concentration following continuous intravenous insulin infusion in dogs was the first evidence suggesting that peripheral insulin can cross the blood–brain barrier (BBB) [17]. The nonlinear correlation between the rise in plasma insulin and CSF insulin levels suggested a saturable means of transport, and thus a receptor-mediated transport system was proposed [18,19,20]. It is plausible that insulin crosses the blood retinal barrier using a similar mechanism to reach the neural retina.

The high levels of insulin in brain extracts, however, pointed towards the de novo synthesis of insulin in the CNS [15]. This was supported by the detection of insulin secretion in neuronal cultures [21], and the presence of insulin immunoreaction within the Golgi and the rough endoplasmic reticulum in the brain [22]. Insulin 2 mRNA was also identified in GABAergic neurogliaform cells in the cerebral cortex of the rat [23] and preproinsulin mRNA was found in rat retinal tissue [24]. Using insulin antisera, insulin immunoreactivity can be detected in the retinal layers including the ganglion cell layer of human and mouse retina, as well as optic nerve glial cells [25]. Whether this small peptide hormone originates peripherally from the pancreatic beta cells and/or is locally produced remains uncertain; nonetheless, insulin signaling was found to play a putative role in CNS neurons.

**Figure 1:**
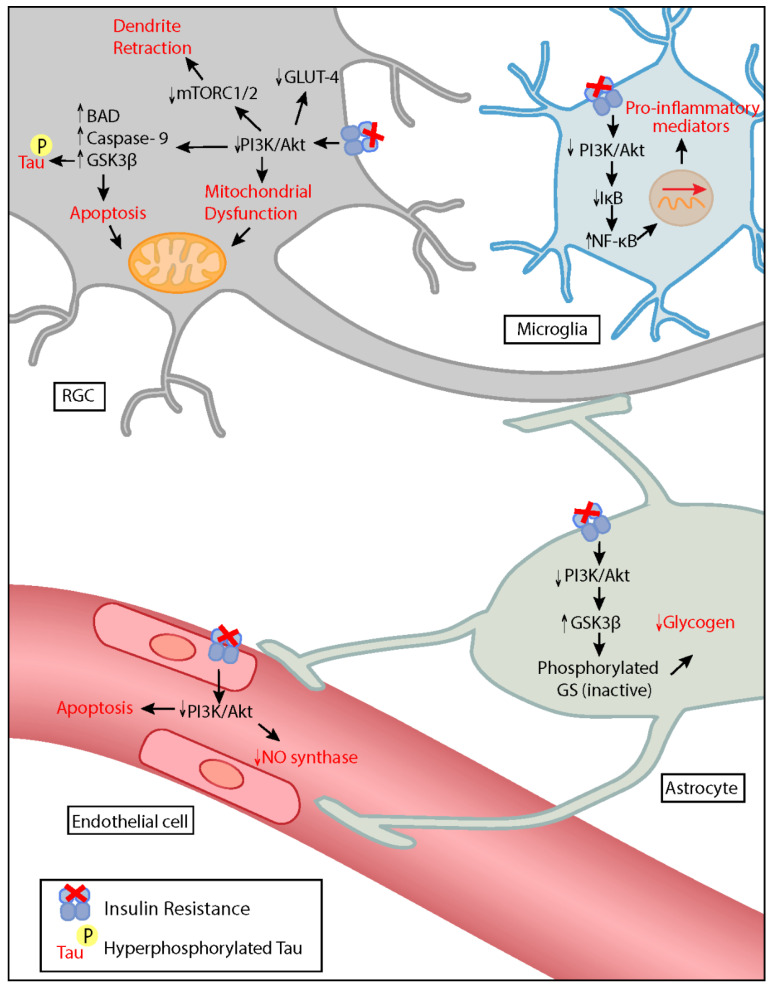
Possible influence of insulin resistance on the pathogenesis of glaucoma. Loss of the insulin signaling pathway reduces activation of Akt pathway. In the RGC, insulin resistance promotes dendritic retraction, mitochondrial dysfunction, tau hyperphosphorylation, and apoptosis. A decrease in insulin signaling in microglia induces the expression of pro-inflammatory mediators. It additionally contributes to vascular dysfunction by causing nitric oxide/endothelin-1 imbalance and endothelial cell apoptosis. In astrocytes, decreased insulin signaling causes depletion of glycogen stores, impairing metabolite redistribution. RGC: Retinal ganglion cell, PI3K: phosphoinositide-3 kinase, Akt: protein kinase B, mTORC: mammalian target of rapamycin complex, Bad: bcl-2 agonist of cell death, GSK3β: glycogen synthase kinase 3- beta, GLUT-4: glucose transporter type 4, NO: nitric oxide, GS: glycogen synthase, IκB: nuclear factor of kappa light polypeptide gene enhancer in B-cells inhibitor, NF-κB: nuclear factor kappa-light-chain-enhancer of activated B cells.

The transduction of insulin signaling is mediated by the binding of insulin to its transmembrane insulin receptor (IR). The insulin receptor is a tyrosine kinase consisting of an α- and β-chain. Binding of insulin causes a conformational change in the β-chain, inducing autophosphorylation of tyrosine residues which in turn trigger downstream events such as the recruitment of insulin receptor substrates (IRS) and other adaptor proteins [26]. Activation of the receptor and the recruitment of adaptor proteins trigger kinase cascades, with two major downstream pathways affected: Raf-1/MEK-MAPK (mitogen associated protein kinase)/ERK (extracellular signal regulated kinase) and the phosphoinositide-3 kinase (PI3K)/protein kinase B (Akt) pathways [27]. These well-established molecular cascades also mediate insulin signaling in the CNS. The downstream effectors of insulin signaling, particularly those of the PI3K/Akt pathway, are expressed in neuronal, glial, and vascular components of the retina and modulate various important functions that are disrupted in glaucoma (Figure 1) [28]. The details of the influence of insulin signaling in the progression of neurodegeneration in glaucoma are discussed below.

## 3. Influence of Insulin Signaling on Glaucoma Pathogenesis

### 3.1. Retinal Ganglion Cell Dysfunction

#### 3.1.1. Apoptosis

Neurodegenerative diseases are characterized by progressive dysfunction and death of specific neuron populations. Disease phenotypes correlate with the functions of the degenerating neuronal population [29]. In AD, memory and cognitive dysfunction occurs as hippocampal neurons undergo apoptosis [30], while in glaucoma, vision loss involves degeneration and eventual apoptotic elimination of RGCs [31,32].

Insulin, well-known for its anabolic function, has recently become known as a neurotrophic factor taking part in neuronal survival and preventing apoptosis. This has been implicated by a substantial number of studies showing that insulin promotes survival following the introduction of different stressors triggering apoptosis. Pre-treatment with insulin prior to the application of hydrogen peroxide and MPP+ neurotoxin decreased reactive oxygen species (ROS) formation and prevented cell death in vitro [33,34]. While serum deprivation induces apoptosis in cortical neurons, the addition of insulin had an anti-apoptotic effect [35]. Correspondingly, insulin deprivation led to the death of external granular layer neurons in rat cerebellar slice cultures [36]. Furthermore, co-injection of insulin and fibroblast growth factor 2 stimulated the proliferation of ciliary marginal zone cells and the production of ganglion cells at the retinal margin in post-hatched chicken [37].

Investigation of the downstream insulin signaling pathways identified PI3K and Akt as central components mediating insulin-induced neuronal survival. Insulin activates PI3K, which then phosphorylates and activates anti-apoptotic substrates. Pre-treatment with PI3K inhibitors prevented protection from serum deprivation-induced cell death [35]. Additionally, activated Akt targets and inactivates pro-apoptotic proteins such as Bad, caspase 9, and glycogen synthase kinase 3 beta (GSK3β). Activated Akt also protects against neuronal hypoxia and nitric oxide (NO)- induced apoptosis by preventing the transcriptional activity of p53 [38].

#### 3.1.2. Mitochondrial Dysfunction

Mitochondria are dynamic organelles that play a vital role in maintaining the high energetic and metabolic demands of the retina. In addition to generating energy in the form of adenosine triphosphate (ATP) via oxidative phosphorylation, these organelles regulate several processes that are essential for neuronal survival such as regulating the production of ROS and apoptosis. Mitochondrial dysfunction has been proposed to play a critical role in the pathogenesis of neurodegenerative diseases. Additionally, the accumulation of mitochondrial DNA mutations and oxidative stress is implicated in ageing, one of the primary risk factors for glaucoma [39]. Hence, the high metabolic demand of the retina paired with the deleterious effects of age-related mitochondrial dysfunction limit the capacity of RGCs to undergo cellular repair, rendering them especially vulnerable to glaucomatous injury [40,41].

Insulin has a stimulatory effect on mitochondrial biogenesis and function. Insulin infusions in human studies increased stimulation of oxidative phosphorylation, promoting the synthesis of ATP and mitochondrial proteins in muscle [42]. These effects are diminished in insulin resistance, as patients with T2DM and high-fat diet fed rats [43] have a reduced capacity to increase muscle ATP production with insulin infusions. Inhibitors of dipeptidyl peptidase-4 (DPP-4) (gliptins) are antidiabetic medications that are used to improve glycemic control in T2DM. Investigations into the effect of Vildagliptin on brain insulin resistance demonstrated its ability to restore neuronal insulin sensitivity; brain mitochondrial function improved, and enhanced cognitive function was observed [44]. Insulin signaling modulates mitochondrial electron transport chain function through the activation of the PI3K/Akt pathway. Insulin inhibits FOXO1/HMOX1 and preserves the NAD^+^/NADH ratio, which regulates the SIRT1/PGC1α pathway for mitochondrial biogenesis and function [45]. This suggests that enhancing mitochondrial function may be an additional therapeutic benefit to targeting insulin signaling in glaucoma.

#### 3.1.3. Dendritic Retraction and Synaptic Impairment

Dendrite retraction is an early pathogenic feature of glaucoma and other neurodegenerative diseases [46,47,48]. Dendrites are delicate projections that receive pre-synaptic inputs from axons and subsequently determine how the neuron will integrate the received information. Pathological disconnection from presynaptic targets leads to significant functional deficit and neuronal death [49]. Therefore, the ability of injured RGCs to regenerate their dendrites may be an important therapeutic strategy in the prevention of synaptic and eventual visual loss in glaucoma. Optic nerve axotomy, resembling glaucomatous axonal injury, results in RGC dendritic structural changes that contribute to neurodegeneration in glaucoma. Systemic or topically administered insulin promotes substantial dendrite, and possibly synapse, regeneration to pre-injury branch length, surface area, and complexity. This was found to be mediated by mTORC1, which controls tree complexity, and mTORC2, which directs dendrite length [50].

Dendritic structural changes may cause abnormal firing patterns of neuronal pathways leading to impairment in synaptic plasticity; a pathologic manifestation implicated in the initial onset of not only glaucoma, but also PD and AD [51,52,53]. Administration of a high-fat diet to induce neuronal insulin resistance in mice causes reduced synaptodendritic protein expression leading to deleterious cognitive effects including impaired working memory [54]. Although the mechanisms that underlie central insulin signaling and synaptic plasticity remain incompletely understood, insulin may have an important role in synaptic plasticity that supports higher brain functions and regulates visual circuit function [55,56,57]. Hence, insulin signaling in glaucoma has the potential to regenerate retracted RGC dendrites and enhance synaptic plasticity.

#### 3.1.4. Tau Hyperphosphorylation

Dysfunctional insulin signaling has been linked to the pathogenesis of aggregated tau neurofibrillary tangles, a major neuropathological hallmark of AD [58,59]. Individuals with glaucoma have been found to have hyperphosphorylated tau in their CSF and ocular samples [60,61,62]. Tau, a microtubule-associated protein abundant in the axon [63,64], plays a critical role in AD and other neurodegenerative diseases, including glaucoma [65,66]. Increasing IOP in a rat glaucoma model exacerbates age-related increase in retinal tau. In RGCs of glaucomatous eyes, tau was depleted from RGC axons in the optic nerve and mislocalized in the dendritic compartment. Tau knockdown using intraocular short interfering RNA, decreased its accumulation in the retina and promoted robust survival of RGCs [67]. These changes support a critical role for tau alterations in ocular hypertension-induced neuronal damage. GSK3β, which normally phosphorylates tau, is inhibited by Akt dependent phosphorylation, a downstream effector molecule in insulin signaling. Therefore, in brain/neuron-specific insulin receptor knockout (NIRKO) mice, the decreased phosphorylation of GSK3β leads to its activation, causing an increase in Tau phosphorylation [68]. This highlights the potential therapeutic role of cerebral insulin in reducing levels of Tau phosphorylation.

#### 3.1.5. Amyloid Deposition

Amyloid-β (Aβ) senile plaques are major pathological hallmarks of AD. Amyloid-β is derived from the amyloidogenic processing of amyloid precursor protein (APP), a neuronal transmembrane protein. This results from the sequential proteolytic cleavage of APP by β- and γ-secretases [69]. APP is synthesized in RGC somas and functions as an important axonal cargo that is transported towards and away from the brain [70]. Early in glaucoma, transport deficits lead to the accumulation of APP [71,72]. In a chronic primate glaucoma model, Aβ was detected in the central visual system, particularly in the lateral geniculate nucleus. These changes were not detected in the hippocampus, which is the most affected brain region in AD [73].

In experimental models of AD, insulin activates insulin degrading enzyme (IDE) which in turn degrades not only insulin but also Aβ [74]. Accordingly, the development of central insulin resistance contributes to the deposition of amyloid in asymptomatic individuals at risk for AD [75,76]. Decreased insulin sensitivity leads to hyperinsulinemia possibly leading to insulin-induced competitive inhibition of IDE activity [77]. This results in impaired degradation of Aβ and promotes AD pathology [78]. Hence, Aβ deposition is a pathological feature of glaucoma that may be a targeted therapeutically via restoration of insulin sensitivity and signaling.

### 3.2. Glial Dysfunction

#### 3.2.1. Neuro-Inflammation

The extracellular milieu, including glial and vascular components, promote neuronal support and survival. However, both components in glaucoma can convert to create a pathogenic extracellular environment for the RGCs and their axons promoting progression of neurodegeneration [2,79]. Astrocytes, microglia and Müller cells are the three major classes of glia in the retina that contribute to the homeostatic environment of the RGC and its response to stressor [80]. The activation of CNS glia has been proposed to take part in age-related neuroinflammation, possibly contributing to neuronal vulnerability with aging and not only triggering, but also driving glaucomatous damage in the retina [81].

Microglial activation precipitating neuroinflammation is implicated in glaucoma pathology [82,83,84]. Treating animal models of glaucoma with minocycline, a tetracycline derivative known to reduce microglia activation, led to decreased microglial activity, improved RGC axonal transport and integrity, and reduced neurodegeneration [85,86]. Insulin can influence the microglial response; insulin resistance and neuroinflammation are two interconnected pathological features of neurodegeneration [87]. In PD, PI3K/MAPK signaling halts microglial activation and prevents the ensuing dopaminergic degeneration [88,89]. Furthermore, the regulation of nuclear factor kappa-light-chain-enhancer of activated B cells (NF-kB), a molecule downstream of the PI3K/Akt pathway, is implicated in the pathogenesis of neuro-inflammation in PD. NF-kB is a transcription factor that regulates the expression of inflammatory genes, mediating the microglial pro-inflammatory response. Activation of the Akt pathway leads to the up-regulation IkB, an inhibitor of NF-kB, resulting in reduced neuroinflammation and enhanced neuroprotection [90,91,92]. Therefore, anti-inflammatory effects of insulin could potentially have a neuroprotective role in glaucoma.

#### 3.2.2. Insulin Action on Astrocytes

Astrocyte glia are present throughout the CNS, including the retina, and have an integral role in neuronal proliferation, axon guidance, neuroprotection, synapse formation/elimination, plasticity, and transmission [93,94,95,96]. Astrocytes are able to exert these effects by forming a cellular network with neurons and other cell types [97]. Bidirectional communication between astrocytes and cells in these networks occurs through connexin-mediated exchange of metabolites, ions, and other small molecules, including energy substrates [98]. Astrocytes are perfectly positioned between the vasculature and neurons to integrate insulin signaling responses in the CNS. Ablation of IRs from astrocytes throughout the CNS significantly reduced glucose transport to the brain, which in turn altered neural activity in the hypothalamus [99], suggesting an important role for astrocytes in insulin signaling.

RGCs, like CNS neurons, have high metabolic energy demands that are primarily met through the utilization of glucose. The expression of the insulin-responsive glucose transporter, GLUT-4, in frog and rat retina, particularly in the ganglion cell layers, suggests that insulin induces glucose uptake in retinal tissue [100]. However, glucose as a source of fuel is not sufficient in times of stress and increased demand, instead astrocytic glycogen stores support and satisfy neuronal metabolic requirements [101,102,103]. Stimulation of astrocytes by insulin promotes glucose uptake and glycogen synthesis [104,105]. These resources are utilized through interactions between astrocytes and neurons during periods of stress, such as neurodegeneration, to promote axon function and neuronal survival [106,107,108]. In glaucoma, metabolites are mobilized through gap junctions composed of connexin 43 (Cx43). Astrocyte networks redistribute these metabolites from the unstressed to the stressed optic projection. This alleviates the bioenergetic stress in the stressed optic nerve, yet it leaves the donating healthy nerve susceptible to metabolic stress [109]. Insulin’s role in stimulating glycogen formation in astrocytes is essential in maintaining stores sufficient to support the metabolic demands of the stressed and unstressed neurons in neurodegenerative diseases. Insulin-like growth factor has been demonstrated to increase Cx43 and gap junctional communication in astrocytes in primary cultures [110]. Thus, the role of insulin signaling in redistribution of metabolites warrants further exploration.

### 3.3. Vascular Dysfunction

Over a range of different ocular perfusion pressures, the retinal and ONH vascular networks maintain a tightly regulated blood supply though endothelial cell- mediated autoregulation of blood flow. This is sustained by a balance between the vasodilator action of NO and vasoconstrictor action of endothelin-1. Vascular dysfunction and breakdown of neurovascular coupling contribute to the pathogenesis of glaucoma [111,112,113]. Endothelial dysfunction underlies the pathogenesis of metabolic syndrome and different vascular disorders including diabetes and hypertension, which share comorbidity with glaucoma [114,115,116,117,118]. Therefore, insulin resistance, which is the hallmark of metabolic syndrome, could potentially underlie the endothelial disturbances that occur in glaucoma. Insulin, via Akt activation, maintains the integrity of the endothelium by preventing endothelial cell apoptosis through the phosphorylation of caspase 9 [119]. Additionally, insulin regulates the balance between NO and endothelin- 1 via PI3K and MAPK dependents signaling in vascular endothelium, respectively [120]. Insulin-mediated activation of endothelial NO synthase in streptozotocin-induced diabetic rats with ischemic stroke provided neuroprotection evident by a decrease in cerebral infarction and neurologic deficits [121]. In insulin-resistant conditions, impairment of the PI3K-dependent signaling may cause imbalance between production of NO and secretion of endothelin-1 and lead to endothelial dysfunction [122]. Hence, improving insulin sensitivity could potentially ameliorate endothelial dysfunction that underlies vascular neurodegeneration in glaucoma.

## 4. Targeting Insulin Resistance Therapeutically in Neurodegeneration

### 4.1. Exogenous Insulin

Restoring insulin signaling in the brain may provide a therapeutic benefit in neurodegenerative diseases. Exogenous insulin can be used to stimulate IRs, inducing downstream molecular pathways. However, the systemic side effects, particularly hypoglycemia, of peripheral intravenous administration of insulin limit its feasibility as a route of administration. Alternatively, the administration of insulin intranasally provides a safe and viable route for delivery of insulin to the CNS without altering blood insulin and glucose levels. This route permits insulin to reach the CSF within minutes as it bypasses the BBB [123]. In animal models of neurodegenerative diseases, exogenous insulin, administered intranasally or topically, demonstrates neuroprotective effects such as promoting neuronal survival, enhancing mitochondrial function, and reducing neuro-inflammation. In models of AD and PD, these effects ameliorate cognitive and motor impairment [124,125,126,127,128,129,130]. Table 1 demonstrates the neuroprotective effects of exogenous insulin use in examples of experimental models of AD, PD, glaucoma, and HIV-associated neurocognitive disorder (HAND).

Investigating the use of intranasal insulin (INI) as a novel treatment option in patients with AD and mild cognitive impairment (MCI) provided clinical evidence of the safety and efficacy of targeting insulin signaling therapeutically in neurodegeneration [131]. For example, administering 20 international units (IU) BID of INI in patients diagnosed with early AD resulted in better verbal memory, improved attention, and functionality 3 weeks following treatment compared to the placebo group [132]. This has also been demonstrated in a number of trials that further revealed positive outcomes and enhanced cognition in AD [133,134,135]. A randomized controlled trial conducted on a small sample of patients with PD (n = 16) evaluated the effects of 40 IU of insulin administered intranasally once daily for four weeks on cognitive and functional performance [136]. The study reported improved verbal fluency and memory, as well as functionality and motor performance. Given the study’s small sample size and short duration, these results warrant further investigation with larger patient cohorts. A recent multicenter phase 2/3 clinical trial investigating use of INI for 12 months in patients with AD and MCI identified no differences in cognitive or functional outcomes between treatment and placebo arms of the study [137]. They did, however, determine that long-term use of INI is safe with no obvious adverse effects; an observation in line with previous literature on the safety of INI use [138].

To our knowledge, no data from clinical trials exist on the utility of exogenous insulin in glaucoma. We need more robust evidence to enable us to translate the use of INI into a new practical approach to preserve RGC function in patients with glaucoma. A study aiming to explore the safety of topical insulin eyedrops in glaucoma is registered on ClinicalTrials.gov (NCT041189200); however, the trial is not yet recruiting. The positive outcomes of the use of exogenous insulin in preclinical and clinical studies and the pathologic similarities between these age-related neurodegenerative diseases make the use of exogeneous insulin a promising potential therapeutic strategy in slowing neurodegeneration in glaucoma.

**Table 1:** Neuroprotective effects of exogenous insulin administration in experimental models of neurodegenerative diseases.

Disease	Experimental Model	Neuroprotective Effect	References
AD	APP/PS1 mice	Reduced β- Amyloid	[124]
	3sxTg-AD mice	Increased synaptic proteins reduced microglia activation	[125]
	ICV-STZ rats	Decreased tau hyperphosphorylation, microglial and astroglia activation, and neuronal loss in the hippocampus	[126,127]
PD	6-OHDA rat	Enhanced mitochondrial function and biogenesis, reduced dompinergic cell death	[128,129,130]
Glaucoma *	Optic nerve axotomy in mice	RGC dendritic regeneration	[50]
HAND	Feline immunodeficiency virus (FIV) infected cats	Reduced glial activation	[139]
	EcoHIV-infected conventional mice	Hippocampal dendritic regeneration	[140]

* Insulin administered topically on the eye; ICV-STZ: intracerebroventricular streptozotocin-injected; 6-OHDA: 6-hydroxylase dopamine; HAND: HIV-associated neurocognitive disorder.

### 4.2. Anti-Glycemic Agents

Enhancing peripheral insulin sensitivity using anti-glycemic agents is one of the first-line management options for the treatment of T2DM. These agents are primarily used to indirectly restore insulin signaling to maintain glycemic control in diabetic patients. Through their therapeutic effects on metabolism and inflammation, all classes of anti-diabetic medications offer beneficial effects in patients with age-related diseases of the CNS. Nonetheless, central insulin resistance is closely associated with the progression of neurodegeneration, thus the most efficacious medications providing the greatest benefit are those that cross the BBB to enhance neuronal insulin signaling.

Metformin is an oral biguanide antidiabetic agent that is being considered for repurposing to treat age-related disorders of the CNS [141]. Through its antioxidant and anti-inflammatory effects, it decreases tau phosphorylation and slows the progression of CNS neurodegeneration [142,143,144]. The use of metformin reduces the risk of glaucoma development in diabetic patients independent of their glycated hemoglobin concentration [145]. These findings could lead to novel treatment in the management of glaucoma if replicated in clinical trials. A clinical trial investigating the effect of metformin on visual function in patients with glaucoma (ClinicalTrials.gov: NCT04155164) is currently recruiting patients. Pre-clinical and clinical studies of the use of other anti-glycemic agents such as thiazolidinediones and GLP-1 analogs in AD and PD also provide promising results [59,77]. Given the epidemiologic and pathologic association between neurodegenerative disease of the CNS and T2DM, anti-glycemic agents are a potential novel treatment option in the management of glaucoma.

## 5. Conclusions

Whether defective insulin signaling directly contributes to the pathogenesis of glaucoma or occurs secondarily as a consequence of neurodegenerative processes remains unclear. Nonetheless, aberrant insulin signaling it is increasingly recognized in its association with neurodegenerative diseases of the CNS. Studying the insulin pathway in RGCs, glial cells, and endothelial cells and understanding the cross talk between insulin signaling and the various pathogenic events in glaucoma are essential to set the stage for future clinical investigations. Several clinical trials that have investigated the potential use of intranasal insulin in the management of AD have revealed positive outcomes that include improved memory and attention. Thus, stimulating insulin signaling directly using exogenous insulin and enhancing its sensitivity using different anti-diabetic medications may have multiple beneficial outcomes. Targeting insulin signaling may serve not only as a possible neuroprotective therapy in glaucoma, but also as a potential pro-regenerative one.
